# Testing a model of adaptation in adolescents and young adults (AYA) with spina bifida (SB)

**DOI:** 10.1186/1743-8454-7-S1-S24

**Published:** 2010-12-15

**Authors:** Kathleen J Sawin, Timothy J Brei, Amy Heffelfinger, Susan E Cashin, Thomas S Webb, Constance F Buran

**Affiliations:** 1 College of Nursing and Children’s Hospital of Wisconsin, UW-Milwaukee, Box 413, Milwaukee, Wi 53201, USA; 2 School of Medicine, Developmental Medicine Department, Indiana University, Indianapolis, INd; Riley Hospital for Children, 702 Barnhill Dr. Indianapolis, IN 46202, USA; 3 Department of Neurology, Division of Neuropsychology, Medical College of Wisconsin, 9200 W. Wisconsin, Ave, Milwaukee, Wi 53226-3596, USA; 4 College of Health Sciences, University of Wisconsin-Milwaukee, Box 413, Milwaukee, Wi, USA; 5 Huntersville Pediatrics and Internal Medicine, Presbyterian Novant Medical Group, 17220 Northcross Dr., Huntersville, NC 28036, USA; 6 Ambulatory Administration, Riley Hospital for Children, 702 Barnhill Dr, ROC 3205 Indianapolis, IN 46202, USA

## Background

Little is known physical health (PH), mental health (MH) and health-related quality of life (HRQOL) adaptation outcomes in AYA with SB, and the factors associated with them. The purpose of this presentation is to 1) describe the PH (functional status [FS], self-management, secondary conditions), MH (depression, overall self-worth, and internal/external behavior problems [IBP, EBP]), and HRQOL outcomes; and 2) delineate the direct and indirect relationships of risk and protective variables in the Ecological Model of Secondary Conditions and Adaptation in SB associated with these outcomes. This model includes SB Context (SES, shunt status and level of lesion [LOL]), neuropsychological risk [NPR], and protective processes (adolescent resilience and family resourcefulness).

## Material and methods

This is an interdisciplinary descriptive cross-sectional study. AYA were between 12-25 years of age and from 4 clinical programs in the USA. They had no other major medical condition, no mental retardation, were English speaking and functioned at approximately grade levels. Parent and AYA report of PH and MH and AYA report of HRQOL outcomes were included. Study measures had reliability and validity data and most had been used in our previous studies. Structural Equation Modelling (SEM) analyses were used to assess the model.

## Results

Mean parent and AYA basic functional status scores reflect need for supervision; self-management reflects need for substantial assistance. Mean parental report of IBP are higher than EBP and both are in the normative range. However, 13-18% of parent or AYA reports of AYA depressive symptoms were in the clinically significant range.

Self-worth and HRQOL were both moderately high. Three SEM analyses revealed different predictive patterns for PH, MH, and HRQOL constructs. In the PH model only LOL was significantly related to FS. NPF mediated the impact of shunt status and LOL on self-management, and age, NPR, and functional status had direct relationships to self-management. In the MH model, SES and family resourcefulness had indirect paths to MH; whereas AYA resilience, secondary conditions, and age had direct paths to MH. In the HRQOL model SES, age, and family resourcefulness had indirect paths to HRQOL through AYA resilience and MH; whereas AYA resilience and MH had direct paths.

## Conclusions

Different direct and indirect relationships of the conceptual framework variables were identified for the three outcomes. Clinical interventions should be differentiated by outcome and targeted to risk and protective variables.

